# Novel loci for triglyceride/HDL-C ratio longitudinal change among subjects without T2D

**DOI:** 10.1016/j.jlr.2024.100702

**Published:** 2024-11-16

**Authors:** Lihua Wang, Siyu Wang, Jason A. Anema, Vaha A. Moghaddam, Yanli Lu, Shiow Lin, E. Warwick Daw, Allison L. Kuipers, Iva Miljkovic, Michael Brent, Gary J. Patti, Bharat Thygarajan, Joseph M. Zmuda, Michael A. Province, Ping An

**Affiliations:** 1 Division of Statistical Genomics, Washington University School of Medicine, St. Louis, MO, USA; 2 Department of Epidemiology, University of Pittsburgh, Pittsburgh, PA, USA; 3 Division of Computation & Data Sciences, Washington University School of Medicine, St. Louis, MO, USA; 4 Department of Chemistry, Washington University School of Medicine, St. Louis, MO, USA; 5 Department of Laboratory Medicine and Pathology, University of Minnesota, Minneapolis, MN, USA

**Keywords:** insulin resistance, aging, genome-wide linkage scan, GWAS, multiomics analyses

## Abstract

Triglyceride (TG)/HDL-C ratio (THR) is a surrogate predictor of hyperinsulinemia. To identify novel genetic loci for THR change over time (ΔTHR), we conducted genome-wide association study (GWAS) and genome-wide linkage scan (GWLS) among nondiabetic Europeans from the Long Life Family Study (n = 1,384). Subjects with diabetes or on dyslipidemia medications were excluded. ΔTHR was derived using growth curve modeling and adjusted for age, sex, field centers, and principal components. GWAS used a linear mixed model accounting for familial relatedness. GWLS employed haplotype-based identity-by-descent estimation with 0.5 cM average spacing. Heritability of ΔTHR was moderate (46%). Our GWAS identified a significant locus at the *LPL* (*P* = 1.58e-9) for ΔTHR; this locus has been reported before influencing baseline THR levels. Our GWLS found significant linkage with a logarithm of the odds exceeding 3 on *3q28* (logarithm of the odds = 4.1). Using a subset of 25 linkage-enriched families, we assessed sequence elements under *3q28* and identified two novel variants (*EIF4A2* [eukaryotic translation initiation factor 4A2]*/ADIPOQ*-rs114108468, *p* = 5e-6, minor allele frequency = 1.8%; *TPRG1*-rs16864075, *p* = 3e-6, minor allele frequency = 8%; accounted for ∼28% and ∼29% of the linkage, respectively). While the former variant was associated with *EIF4A2* (*p* = 7e-5)/*ADIPOQ* (*P* = 3.49e-2) transcriptional levels, the latter variant was not associated with *TPRG1* (*P* = 0.23) transcriptional levels. Replication in the Framingham Heart Study Offspring Cohort observed modest effect of these loci on ΔTHR. Our approach discovered two novel gene variants *EIF4A2/ADIPOQ*-rs114108468 and *TPRG1*-rs16864075 on *3q28* for ΔTHR among subjects without diabetes. Our findings provided novel insights into the molecular regulation of insulin resistance.

Triglyceride (TG)/HDL-C ratio (THR) is an accurate surrogate marker of insulin resistance (IR), where a higher THR indicates a poor response of cells in muscles, fat, and liver to insulin (1). IR is a well-known underlying pathogenesis of T2D. T2D affects over half a billion people globally with its complications influencing cardiovascular system, kidney, and neurological organs (2). In the United States, the total cost of diabetes in 2022 was $412.9 billion including $306.6 billion of direct medical costs and $106.3 billion of indirect costs (3). To reduce the medical costs of diabetes, prevention is the most efficient strategy. Commonly recommended prevention approaches of diabetes include several lifestyle changes (eating healthy diet, exercising regularly, and avoiding tobacco use). However, given a 3% increase in the national prevalence of diagnosed diabetes from 2017 to 2022 (3), these lifestyle changes are apparently not sufficient to prevent diabetes. Accurate markers to identify high-risk individuals decades before the onset of diabetes, along with early intervention, could lead to promising outcomes in disease prevention. THR is one such marker that can predict prediabetes. As such, disentangling the genetic of THR in nondiabetics will unveil molecular mechanisms of IR, nominate potential diagnostic markers that distinguish high-risk from low-risk individuals, as well as provide novel therapeutic targets to high-risk individuals. The largest genome-wide association study (GWAS) of THR to date has been conducted and identified 369 independent SNPs using a sample of 402,398 unrelated Europeans (4). However, this GWAS analysis is confined to examining cross-sectional THR at one time point in unrelated samples, lacking the statistical power to unveil genetic variants contributing to the longitudinal change of THR (hereafter referred to as ΔTHR), especially in information-rich family data. ΔTHR reflects variation of THR level within months or years, which cannot be captured by THR measured at a single time point. For instance, a normal THR level at a single time point cannot tell whether the THR level is stable or on a steadily increasing trend. Rising THR over time (i.e., higher ΔTHR) indicates a greater risk for IR. Consequently, there might be different genetics regulating ΔTHR. However, the genetic determinants of ΔTHR in family-based cohorts remains elusive.

In this study, our first goal is to detect genetic variants regulating ΔTHR in a family-based cohort. We utilized the NIA Long Life Family Study (LLFS) in the discovery phase. LLFS, a multicenter family-based cohort, measured THR in two visits spaced 5–7 years apart (5). Two orthogonal analyses including GWAS (adjusting familial relatedness) and genome-wide linkage scan (GWLS; modeling familial relatedness) were employed to maximize our statistical power. GWAS aims to identify the associations of the variants with the traits in a large population. In family-based cohorts, GWAS estimates the influence of the variants on the traits as a fixed effect correcting for a random effect of relatedness among family members. Whereas GWLS examines the familial relationship to detect the genetic region or variants linked to the traits. To demonstrate the biological relevance of the variants identified by GWAS and GWLS, our second goal is to map the variants to the functional molecules (RNA transcripts and lipids) utilizing blood samples of LLFS. Framingham Heart Study Offspring Cohort (FOS) was used to replicate the variants identified in the discovery phase.

## Materials and Methods

### Cohort demographics, phenotype preparation, multiomics assays, and statistical analysis for LLFS participants

#### Cohort demographics

LLFS is a multinational, multicenter, and multigenerational family-based longitudinal study that recruited exceptional long-lived pedigrees. LLFS, predominantly of European descent, constitutes a total of four field centers including Boston University Medical Center in Boston (MA), Columbia College of Physicians and Surgeons in New York (NY), the University of Pittsburgh in Pittsburgh (PA), and the University of Southern Denmark field center (5). To estimate ΔTHR using random coefficient model (6), we excluded subjects with diabetes or those taking medications for dyslipidemia at either visit 1 or visit 2 and used 1,384 nondiabetic participants with TG and HDL-C assayed at both visit 1 and visit 2 (Table 1). Among 1,384 samples, about 39.4% (545) are from the Denmark center, 25.8% (358) from the Boston center, 20.4% (282) from the Pittsburgh center, and 14.4% (199) from the Columbia center. The studies in this work abide by the Declaration of Helsinki principles. The protocols were approved by the Institutional Review Board of each study center.

**Table 1:** Demographic information of samples by field centers

Demographic categories	All	Boston	Columbia	Denmark	Pittsburgh
N	1,384	358	199	545	282
Female (%)	821 (59.3)	220 (61.5)	120 (60.3)	299 (54.9)	182 (64.5)
Age (mean ± SE)	61.9 ± 0.3	60.8 ± 0.6	63.6 ± 0.9	61.7 ± 0.4	62.6 ± 0.8
TG (mean ± SE)	101.23 ± 1.7	101 ± 3.6	101. ± 5.7	104. ± 2.7	95.4 ± 2.8
HDL-C (mean ± SE)	63.54 ± 0.5	63.3 ± 1.0	63.4 ± 1.3	64.3 ± 0.7	62.5 ± 1.0
ΔTHR (mean ± SE)	0.0 ± 2.7	−0.1 ± 2.8	−0.5 ± 2.8	0.4 ± 2.7	−0.2 ± 2.7

ΔTHR, longitudinal change of TG/HDL-C estimated with values from visits 1 and 2.

Boston = Boston University; Columbia = Columbia University; Denmark = University of Southern Denmark; Pittsburgh = University of Pittsburgh.

Age is age in years at baseline visit 1; values denote mean ± SE.

TG denotes triglyceride assayed in mg/dl at baseline visit 1.

#### Phenotype preparation

In LLFS, blood was drawn after at least 8 hours of fast, and blood TGs (mg/dl) and HDL-C (mg/dl) were quantified by the LLFS’s central laboratory at the University of Minnesota (7). TG levels were determined using the glycerol-blanked method with the Roche Diagnostics system, while HDL-C was assessed using the third-generation HDL-C test also from Roche Diagnostics. Both visit 1 and visit 2 employed the same protocol for assaying TG as well as HDL-C. The lab assays performed at two distinct time points spaced 7 years apart for the same subset of LLFS samples that showed strong correlated values (Pearson’s correlation coefficient r = 0.62 for TG and r = 0.78 for HDL). THR was calculated for LLFS visit 1 and visit 2 separately and followed by logarithmic transformation to approximate a normal distribution. We further created THR residuals by forcing baseline age, field centers, sex in the model, and stepwise regressing out 10 principal components (PCs) (see *Whole Genome Sequencing* [WGS] section for details) for both visit 1 and visit 2. To estimate the random slope (ΔTHR) for each LLFS participant, we treated visit 1 as baseline (years = 0) and calculated time passed at visit 2 as years = (visit 2 date-visit 1 date)/365.25. Since THR residuals of two visits for each participant are not independent, we assumed an unstructured covariance matrix among two visits (type = un) and estimated ΔTHR as the random slope by fitting years against THR residuals using a linear mixed model in SAS 9.4 (proc mixed method = ml). This linear mixed model can be expressed as THRres_ij_ = β_0_+ β_1_∗years_ij_ + u_0j_ + u_ij_∗years_ij_+ε_ij_ (THRres_ij_ denotes the THR residual for jth individual and ith visit, β_0_ is the intercept for LLFS participants, β_1_ is the average change per year, u_0j_ is the random intercept for jth individual, u_ij_ is the random slope for jth individual, ε_ij_ is the residual error at ith visit for individual j, u_0j_ ∼ N(0, τ^2^), and ε_ij_ ∼ N(0, σ^2^). The details of this growth curve modeling were described by Corbett *et al.* (6). Family relatedness was not considered in the estimation of ΔTHR here and was adjusted in the association analyses.

#### Multiomics assays in LLFS

##### Whole Genome Sequencing

WGS for LLFS participants in 150 bp paired-end reads was performed using Illumina sequencer by the McDonnell Genome Institute (MGI) at Washington University in Saint Louis. MGI also conducted reads alignment to Genome Reference Consortium Human Build 38 (GRCh38) with Burrows-Wheeler Aligner (BWA-MEM 0.7.15), marking duplicates with Picard 2.4.1, base quality score recalibration with GATK BaseRecalibrator 3.6, and lossless conversion to CRAM format with SAMtools 1.3.1. The variant calling was carried out at the Division of Statistical Genomics at Washington University in St. Louis (8). The four steps of variant calling are *1*) CRAM files were converted to subject-level GVCF files with GATK HaplotypeCaller; *2*) GVCF files were combined using GATK CombineGVCF; *3*) Genotype of identified variants were called with GATK GenotypeGVCFs; and *4*) the diallelic SNPs were extracted using GATK SelectVariants. The additional quality control (QC) procedures include exclusion of contaminated samples with FREEMIX >0.03, samples with <20x of haploid coverage, as well as samples with high Mendelian errors reported by LOKI 2.4.5 (9) and KING 2.3.1. The low quality variant site that had depth <20 or >300, Hardy-Weinberg equilibrium *p* < 1e-6, or heterozygosity >0.55 were removed. The sample swap was identified and corrected by comparing WGS against GWAS chip data. After QC, 1,720 participants with 32,892,186 autosome diallelic SNPs remained, and 1,384 samples with available ΔTHR data were included in our analyses. PCs were estimated using Eigenstrat (10).

##### RNA-Seq For Blood Samples at LLFS Visit 1

MGI extracted RNA in LLFS participants from the PAXgene™ Blood RNA tubes using the Qiagen PreAnalytiX PAXgene Blood miRNA Kit (Qiagen, Valencia, CA). The whole blood paired-end RNA-Seq library prep, QC, alignment, and gene expression matrix were done by the Division of Computation & Data Sciences at Washington University (11). The nf-core/rnaseq 3.14.0 was employed to obtain the quantification of the RNA-Seq data (https://nf-co.re/rnaseq/3.14.0). The major steps involve aligning the reads to GRCh38 with STAR, marking read duplicates with Picard MarkDuplicates, and transcript assembly and quantification with StringTie. We additionally removed the genes with fewer than four counts per million in at least 98.5% of samples as well as the genes with more than 8% of intergenic overlap. After QC, 1,810 samples with 16,418 genes remained, and 654 samples with available ΔTHR data were utilized in our study. The count of each gene was normalized using a variance stabilizing transformation from the fitted dispersion-mean relation in DESeq2.

##### Measurement of Blood Lipid Metabolites at LLFS Visit 1

The untargeted metabolomics workflow for lipids in LLFS participants was conducted at the Biomedical Mass Spectrometry Lab at Washington University (12). In brief, lipid metabolites were extracted from plasma samples using a solid-phase extraction plate with methyl tert-butyl ether/methanol. Subsequently, the lipid extract was dried using nitrogen flow. The *m/z* values of lipid metabolites were obtained via reversed-phase chromatography coupled to high-resolution mass spectrometry in positive mode. Lipid Annotator was employed for annotating the lipid iterative MS/MS data. The peak areas of the annotated peak list were quantified using Skyline (13) (version 20.1.0.155). Blank samples were introduced at the beginning and end of each batch to detect background peaks. To mitigate batch variation, a pooled QC sample served as an internal standard, and a random forest-based method was applied to correct peak areas (14). After QC, 188 lipids from 13 compound classes remained.

#### Statistical analyses

##### GWAS Using WGS Data

To identify the sequenced variants that are associated with ΔTHR, we conducted GWAS analyses using a linear mixed model for additive dosage of the variants. We defined the major allele of each variant as the reference allele (REF). For instance, the dosage is 0 for genotype of two copies of reference allele (REF/REF), 1 for one copy of reference and one copy of alternative allele (REF/ALT), and 2 for two copies of alternative allele (ALT/ALT). If a variant has two alleles “A” and “C,” the allele “A” is the major and reference allele. We coded the dosage of this variant as “0” for “AA” genotype, “1” for “AC” genotype, and “2” for “CC” genotype. The dosage of this variant will be used as an independent variable. To account for the familial relationship, the kinship matrix was estimated using “kinship” R package. A linear mixed model implemented in “lmekin” R package was utilized to estimate the fixed effect of additive dosage of the variants (independent variable) for ΔTHR (dependent variable) accounting for the random effect of kinship matrix. The statistical equation for this linear mixed model is ΔTHR = β∗SNP + u∗Z+ε (Z denotes the kinship matrix, u indicates the random effects, and ε is independent error terms). The significant variants were identified with *p* < 5e-8. The Manhattan plot and quantile-quantile plots were generated using “qqman” package in R. The *r*^2^ measure of linkage disequilibrium (LD) was estimated in Haploview4.1.

##### GWLS and Fine-Mapping Under the Linkage Peak

To detect the genetic regions contributing to heritability of ΔTHR within families, we first selected up to five tightly linked SNPs within each of 0.5 centiMorgan (cM) interval across the autosome and built information-rich haplotypes using ZAPLO (15). From these haplotypes, we estimated multipoint identity-by-descent using LOKI 2.4.5 (9) in intact pedigrees and performed GWLS via Sequential Oligogenic Linkage Analysis Routines (SOLAR) (16). Any autosomal region with logarithm of the odds (LODs) above 3 was identified as significant linkage peak and selected for further analysis. As ΔTHR is a complex trait potentially influenced by multiple genes and their interactions, variation of ΔTHR among different families may be attributed to different genetic factors and regions. Consequently, due to genetic heterogeneity and varying penetrance across families, only selected LLFS families had pedigree-specific LODs exceeding 0.1 and contributed to the significant linkage peak. The LODs estimated using these selected families were defined as heterogeneity LOD (HLOD). To enhance the statistical power of our fine-mapping and nominate potential driver SNPs, we focused on these selected families. Utilizing these families and the same linear mixed model as described in GWAS, we first identified the top sequenced variants under the linkage peak that were associated with ΔTHR. To quantify the linkage peak explained by the top sequenced variants, we generated a dataset comprising ΔTHR and dosage with identical nonmissing patterns for each of these variants. Next, we assessed the HLOD prior to SNP adjustment in SOLAR without any covariates, then obtained the HLOD after SNP adjustment by integrating the dosage of each SNP as a covariate in SOLAR. We subsequently calculated the percentage of linkage explained by the difference in HLOD before and after SNP adjustment divided by the HLOD before adjustment.

##### Expression Quantitative Trait Loci Analysis in LLFS

To map the sequenced variants to their regulated transcripts, we utilized visit 1 LLFS RNA-Seq data and first adjusted normalized values of their nearby genes by age, sex, field centers, white blood cell count, red blood cell count, platelets, monocyte, neutrophils, plates, percent of intergenic reads, and 10 PCs and then tested the association of the sequenced variants with the adjusted residuals using a linear mixed model implemented in “kinship” and “lmekin” R package. The statistical equation for this linear mixed model is RNAres = β∗SNP + u∗Z+ε (Z denotes the kinship matrix, u indicates the random effects, and ε is independent error terms).

##### Metabolite Quantitative Trait Loci Analysis in LLFS

The variants influencing ΔTHR may operate through the regulation of blood levels of lipid metabolites. To assess the association of sequenced variants with lipid metabolite level in blood during the first visit of LLFS, we log2-transformed the peak area of each lipid metabolite to approximate a normal distribution. Subsequently, we adjusted the transformed values for the effects of age, sex, field centers, and 10 PCs. The effect size of the sequenced variants on the adjusted residuals was determined using a linear mixed model implemented in “kinship” and “lmekin” R package. The statistical equation for this linear mixed model is Lipidres = β∗SNP + u∗Z+ε (Z denotes the kinship matrix, u indicates the random effects, and ε is independent error terms).

### Cohort Demographics and Replication Analysis Using FOS Participants

To replicate our discovery findings in both GWAS and linkage region, we used the FOS data (https://www.framinghamheartstudy.org/). FOS is a longitudinal population-based study of Framingham (MA) residents (17, 18). Our replication utilized the 859 samples from offspring cohorts who had TG and HDL-C assayed in both examination 5 (average age: 54.63 ± 9.80) and 7 (average age: 61.54 ± 9.76). In FOS, TG levels were measured using automated enzymatic assay procedures, and HDL-C was quantified using dextran sulfate-Mg^2+^ precipitation procedure (19). Participants with diabetes or on medications for dyslipidemia at either examination 5 or 7 were excluded from our replication. As part of the National Heart, Lung and Blood Institute’s TOPMed phase I, WGS data from FOS were sequenced at >30× depth of coverage from the Broad Institute of the Massachusetts Institute of Technology and Harvard. The joint calling of all samples along with QC of variants and samples was performed by the TOPMed Informatics Resource Center at the University of Michigan (20). THR was calculated for nondiabetic participants who are not on dyslipidemia medication and had TG and HDL-C levels from both FOS examination 5 and 7. After obtaining the residuals adjusted for baseline age and sex, ΔTHR in FOS was estimated in a linear mixed model via SAS 9.4. To replicate the association of variants detected in LLFS, the same linear mixed model implemented in “kinship” and “lmekin” R package as described above was used.

## Results

### Characteristics of LLFS samples

As shown in Table 1, each of the four field centers included more than half the female participants: 64.54% (182) from the Pittsburgh center, 61.45% (220) from the Boston center, 60.3% (120) from the Columbia center, and 54.86% (299) from the Denmark center. The mean age of blood draw in the first visit of these 1,384 individuals is 61.9 years old, ranging from 60.8 in the Boston center to 63.6 in the Columbia center. The average TG level of 1,384 individuals is 101.23 mg/dl. Samples from the Pittsburgh center had relatively low levels of TG (95.4 mg/dl) than participants from the Boston center (101 mg/dl), Columbia center (101 mg/dl), and Denmark center (104 mg/dl). The mean HDL-C level of all samples is 63.54 mg/dl and varies slightly from 63.3 mg/dl in Boston center, 63.4 mg/dl in Columbia center, 64.3 mg/dl in Denmark center, and 62.5 mg/dl in Pittsburgh center. The mean ΔTHR level ranges from −0.5 in Columbia center to 0.4 in Denmark center (Table 1).

### Discovery of *LPL* locus from GWAS using LLFS samples

**Fig. 1:**
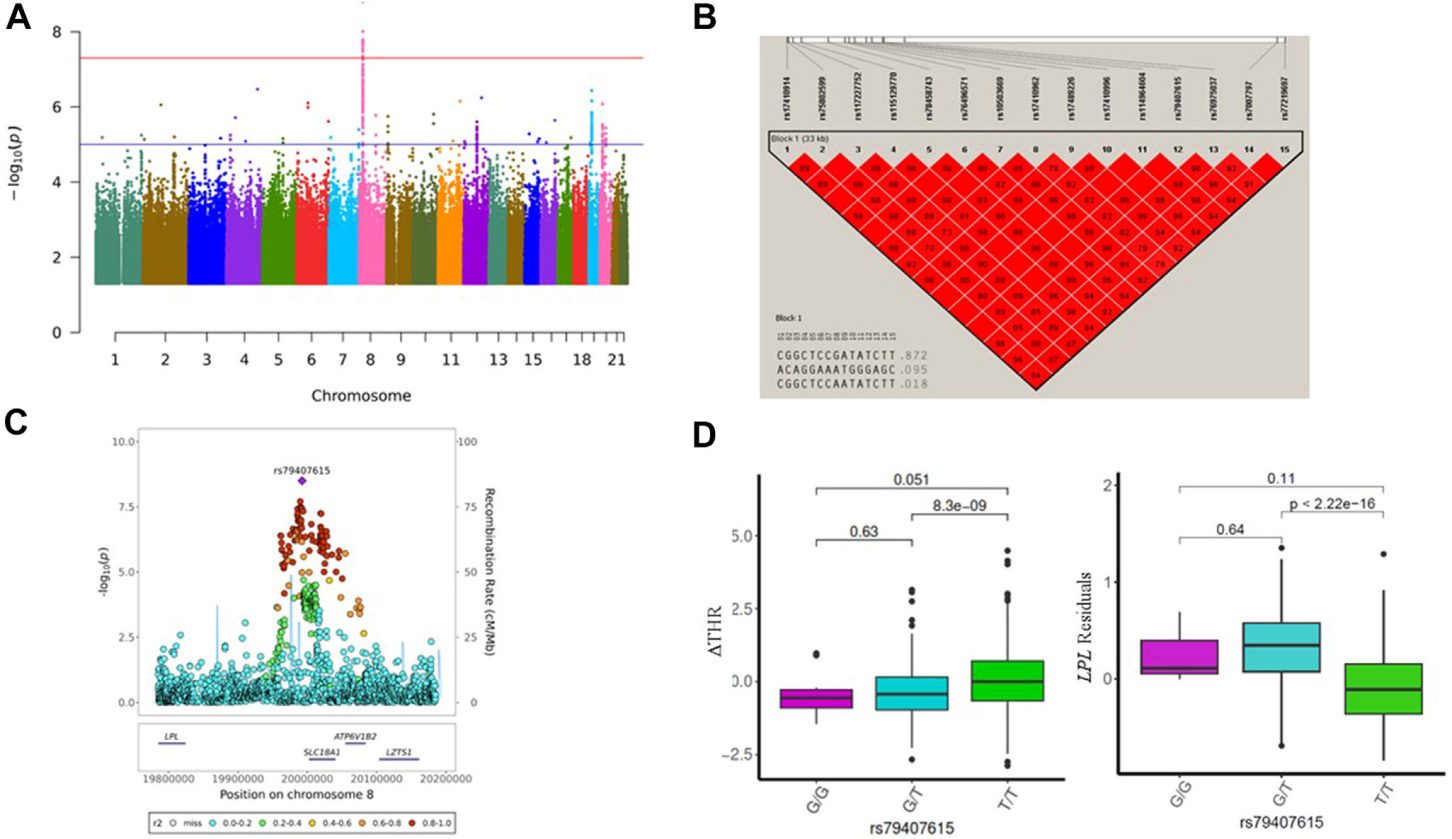
GWAS results of ΔTHR using whole genome-sequenced autosome variants. A: The Manhattan plot for GWAS results of ΔTHR across 22 chromosomes. *P* values are two-sided raw *P* values estimated from a linear additive model. The *y*-axis depicts the negative log10-transformed *P* value. The *x*-axis is genomic coordinates by chromosome number. The blue-colored solid horizontal line denotes the suggestive threshold (*p* = 1e-5). The red-colored solid horizontal line indicates the genome-wide significant cutoff at *p* = 5e-8. B: The LD heatmap of 15 significant SNPs at chromosome 8 that reached genome-wide significance using Haploview. The value displayed is LD r2. C: The Locuszoom plot of ± 200 Kb of lead SNP rs79407615 at chromosome 8. The *x*-axis is the base pair position in the genome build GRCh38 at chromosome 8. The *y*-axis is the –log10 of the two-sided *P* value for each genetic variants. D: The box and whisker plot for the ΔTHR and the *LPL* residuals across three genotypes of rs79407615. The pairwise comparison *P* value is estimated using wilcox.test in R.

For the 1,384 LLFS samples, we conducted a GWAS of ΔTHR using 7,944,836 sequenced SNPs with minor allele frequency (MAF) greater than 0.01. Fifteen common variants, located at 18,692 bp–52,691 bp downstream of *LPL* at chromosome 8, reached the genome-wide significant threshold (*p* < 5e-8; Figure 1A,C). Based on Haploview, these 15 variants are in LD with each other, all with pairwise *r*^2^ > 0.8 (Fig. 1B). The SNP rs79407615 is the sentinel variant of the identified region. We found that the G allele of rs79407615 is notably enriched in our samples (G = 9.44%), which is almost four times the frequency of this allele in the National Center for Biotechnology Information Allele Frequency Aggregator (ALFA) European database (G = 2.38%). The participants carrying G allele of rs79407615 had lower ΔTHR (β = −0.4123; *P* = 1.58 × 10^-9^; Fig. 1D), indicating protective role of this SNP against the development of IR and T2D. In addition, the G allele of rs79407615 was also associated with lower THR in LLFS visit 1 (β = −0.37; *P* = 4.65 × 10^-8^). To examine whether *LPL* is the functional molecule that mediates the effect of this SNP, we examined the association of rs79407615 with blood expression of *LPL*. We observed that the G allele of rs79407615 was associated with significantly higher blood *LPL* expression (β = 0.3233; *P* = 3.004e-43; Fig. 1D), highlighting *LPL* is the molecule that links rs79407615 to ΔTHR.

### Validation of previous reported loci influencing cross-sectional THR

Oliveri *et al*. (4) reported 369 independent SNPs for cross-sectional THR in a GWAS study using 402,398 Europeans. About 96% (354/369) of these SNPs are available in LLFS WGS data. Among these 354 SNPs, we found 25 SNPs (7.06%) from 23 loci (*LPL*, *ANGPTL3*, *ANGPTL4*, and *TM6SF2*, as well as others), had *P* < 0.05, and showed the same direction of effect for ΔTHR (Table 2). Notably, the SNP rs268, a missense variant of *LPL* (MAF = 1.82%; β = 0.58; *P* = 1.11e-4), passed the Bonferroni-corrected threshold (0.05/354 = 1.27e-4) for association with ΔTHR in LLFS, and its G allele led to a higher THR (β = 0.29; *P* = 1.11e-136; Oliveri’s paper) and a higher ΔTHR (LLFS).

**Table 2:** Validation of reported loci for cross-sectional THR

rsID	Chr	Position	Locus	Role	CA	Non_CA	EAF	LLFS			Oliveri *et al.*		
								β	SE	P	β	SE	P
rs10889333	1	62491359	*ANGPTL3*	Intronic	A	G	0.328	−0.124	0.043	3.70E-03	−0.066	0.003	3.37E-89
rs13094241	3	196464022	*UBXN7-AS1*	Intergenic	T	G	0.262	0.096	0.046	3.61E-02	0.021	0.003	5.11E-09
rs998584	6	43790159	*VEGFA*	Intergenic	A	C	0.489	0.088	0.039	2.44E-02	0.062	0.003	8.55E-86
rs12208357	6	160122116	*SLC22A1*	Nonsynonymous_SNV	T	C	0.068	0.168	0.078	3.04E-02	0.042	0.005	1.44E-11
rs17145750	7	73612048	*MLXIPL*	Intronic	T	C	0.167	−0.110	0.054	4.28E-02	−0.142	0.004	2.76E-241
rs111914893	7	74587994	*GTF2IRD1*	Intronic	T	C	0.062	0.226	0.081	5.16E-03	0.041	0.006	1.51E-08
rs10260148	7	130746210	*KLF14*	Intergenic	T	C	0.268	0.114	0.045	1.07E-02	0.043	0.003	9.46E-35
rs62492368	7	150840547	*AOC1*	Intergenic	A	G	0.320	0.099	0.042	2.02E-02	0.022	0.003	8.89E-11
rs7012814	8	9315848	*LOC157273*	Intergenic	A	G	0.468	−0.113	0.040	5.35E-03	−0.038	0.003	2.07E-33
rs268	8	19956018	*LPL*	Nonsynonymous_SNV	G	A	0.018	0.584	0.151	1.11E-04	0.292	0.010	1.11E-136
rs73667496	8	20080341	*LPL*, *SLC18A1*	Intergenic	T	C	0.060	−0.232	0.084	6.04E-03	−0.099	0.006	6.59E-44
rs7015766	8	20081538	*LPL*, *SLC18A1*	Intergenic	T	C	0.076	−0.251	0.077	1.13E-03	−0.263	0.005	8.39E-405
rs6999569	8	125463528	*TRIB1*	Intergenic	G	A	0.488	−0.106	0.041	9.19E-03	−0.108	0.003	6.83E-258
rs9919491	10	92995002	*EXOC6*	Intronic	A	T	0.275	0.103	0.047	2.98E-02	0.021	0.003	2.64E-09
rs480823	11	116655013	*LINC02702*	NcRNA_intronic	C	T	0.083	0.262	0.071	2.44E-04	0.206	0.005	2.09E-267
rs117233107	12	4219355	*CCND2*	Intergenic	A	G	0.019	−0.525	0.146	3.32E-04	−0.088	0.011	9.84E-11
rs72735627	15	40765309	*GCHFR*	UTR3	T	C	0.120	−0.149	0.062	1.70E-02	−0.032	0.004	1.85E-09
rs139974673	15	43735687	*MAP1A*	Downstream	C	T	0.034	0.296	0.112	8.27E-03	0.197	0.008	2.51E-88
rs72786786	16	56951602	*HERPUD1*	Intergenic	A	G	0.331	−0.090	0.041	2.94E-02	−0.151	0.003	4.12E-421
rs7499892	16	56972678	*CETP*	Intronic	T	C	0.169	0.162	0.053	2.33E-03	0.171	0.003	2.15E-381
rs931992	17	39665182	*STARD3*	Upstream; downstream	G	T	0.344	0.096	0.042	2.28E-02	0.020	0.003	8.65E-10
rs116843064	19	8364439	*ANGPTL4*	Nonsynonymous_SNV	A	G	0.024	−0.441	0.131	7.67E-04	−0.260	0.009	5.57E-116
rs58542926	19	19268740	*TM6SF2*	Nonsynonymous_SNV	T	C	0.081	−0.224	0.073	2.14E-03	−0.108	0.005	7.28E-74
rs185799410	20	58891038	*GNAS*	Intronic	T	G	0.028	0.263	0.121	3.02E-02	0.056	0.008	2.57E-08
rs2223041	21	15050282	*NRIP1*	Intronic	T	C	0.386	0.123	0.042	3.74E-03	0.018	0.003	5.00E-08

β, the effect estimate; CA, the allele used to estimate the effect; Chr, chromosome number; EAF, the frequency of coded allele; Locus, mapped gene locus; Non_CA, the other allele not used for estimation of the effect; pos, base pair position.

### GWLS and fine mapping detected *EIF4A2/ADIPOQ*-rs114108468 and *TPRG1*-rs16864075 at 3q28

**Fig. 2:**
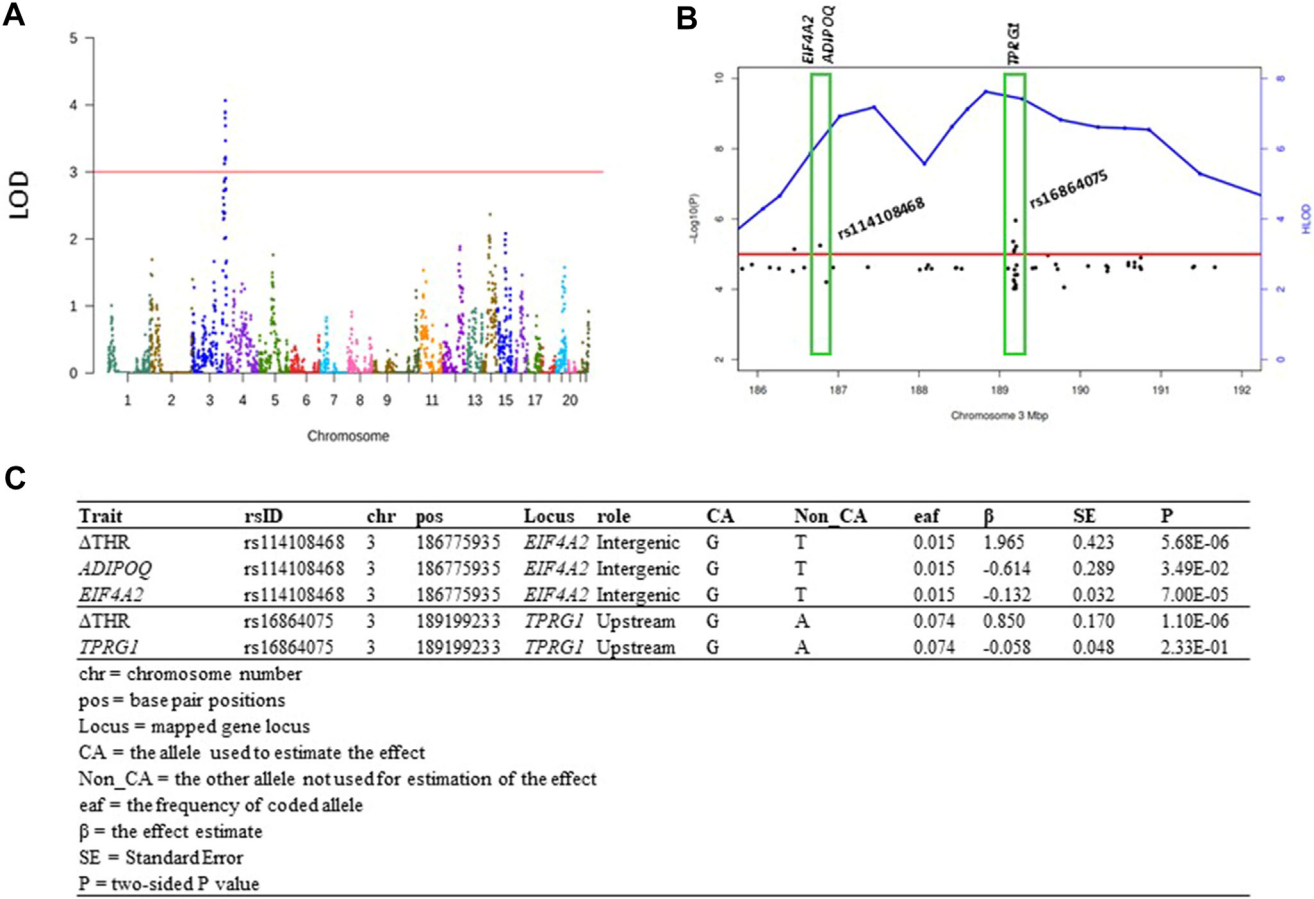
Genome-wide linkage analyses of the ΔTHR identified a linkage peak at chromosome 3. A: The plot of the LOD score across 22 autosomes. The *y*-axis is the LOD score estimated with SOLAR. The *x*-axis is the genomic coordinates by chromosome number. B: The plot of negative log10-transformed *P* value (*y*-axis at left side) and HLOD (*y*-axis at right side) versus physical position in Mbp at chromosome 3. C: The association results of rs114108468 and rs16864075 with ΔTHR, *ADIPOQ*, *EIF4A2*, and *TPRG1*.

In LLFS, heritability of THR at two visits (40% in visit 1 and 32% in visit 2) was comparable to ΔTHR (46%). Our GWLS, conducted using SOLAR, identified a genomic region located at 3q28 with LOD score exceeding 3.0 (LODs = 4.1; Fig. 2A). At this linkage peak (184,989,149 bp—192,579,505 bp), we selected 25 families encompassing 234 participants whose pedigree-specific LODs exceeded 0.1. Using these selected families, the HLOD score collectively reached 6.95 at 197 cM, significantly higher than the LOD score obtained from the analysis of 397 pedigrees. To pinpoint the genetic variants contributing to the linkage peak, we initially investigated the association of sequenced variants in the region with the ΔTHR of these 25 families using a linear mixed model and followed by fine mapping. As shown in Fig. 2B, we detected two variants, rs114108468 (133,231 bp upstream of *ADIPOQ*; 13,962 bp downstream of *EIF4A2* [eukaryotic translation initiation factor 4A2]) and rs16864075 (an intronic variant of *TPRG1*), which had *P* < 1.0e-4 for ΔTHR. These two SNPs are independent and not in LD with each other (*r*^2^ = 0.0003). The SNP rs114108468, a rare variant, had enriched G allele (G = 1.5%) in our selected families than in ALFA European database (G = 0.7%), and dosage of G allele correlated with a higher ΔTHR (β = 1.965; *P* = 5.68e-6; Fig. 2C). The second SNP rs16864075 also showed higher G allele frequency (G = 7.38%) in our samples than in ALFA European database (G = 6.11%), and this G allele significantly increased the ΔTHR (β = 0.85; *P* = 1.10e-6; Fig. 2C). Further linkage analysis for fine mapping, conditioning on each of rs114108468 and rs16864075, revealed an HLOD drop of 1.94 (∼28% of linkage) by rs114108468 and 2.05 (∼29% of linkage) by rs16864075, supporting their significant contribution to the linkage peak. Subsequently, integration of blood transcriptomic data uncovered that the G allele of rs114108468 is associated with a significantly lower *ADIPOQ* level (β = −0.6134; *P* = 3.49e-2; Fig. 2C) and lower *EIF4A2* level (β = −0.1322; *P* = 7.00e-5; Fig. 2C) in the blood of LLFS participants, implicating *ADIPOQ* and *EIF4A2* in the regulation of ΔTHR. No notable association was found between rs16864075 and *TPRG1* (*P* = 2.33e-1).

### Multiple lipids associated with rs79407615, rs114108468, and rs16864075

TG is one of the components involved in the calculation of THR. To test whether the *LPL* locus and 3q28 locus harbors metabolite quantitative trait loci of lipids, we examined the association of rs79407615, rs114108468, and rs16864075 with 188 lipids from blood at visit 1. As shown in Table 3, 14 lipids from two compound classes (diacylglycerol and triacylglycerol) for rs79407615, 22 lipids from four compound classes (phosphatidylethanolamine, diacylglycerol, triacylglycerol, and cholesteryl ester) for rs114108468, and nine lipids from three compound classes (diacylglycerol, triacylglycerol, and cholesteryl ester) passed the Bonferroni-corrected threshold (*P* < 2.66e-4), supporting the involvement of *LPL* locus and 3q28 locus in the regulation of multiple lipids.

**Table 3:** Lipids associated with *LPL* locus and 3q28 locus

rsID	Chr	Lipids	Compound class	CA	Non_CA	N	β	SE	P
rs79407615	8	DG.36.3	Diacylglycerol	G	T	1,216	−0.221	0.045	9.22E-07
	8	DG.34.2	Diacylglycerol	G	T	1,216	−0.218	0.048	6.42E-06
	8	DG.36.2	Diacylglycerol	G	T	1,216	−0.215	0.048	7.02E-06
	8	TG.53.3	Triacylglycerol	G	T	1,216	−0.119	0.028	1.84E-05
	8	DG.34.1	Diacylglycerol	G	T	1,216	−0.226	0.053	2.06E-05
	8	DG.34.3	Diacylglycerol	G	T	1,216	−0.188	0.045	2.60E-05
	8	DG.36.4	Diacylglycerol	G	T	1,216	−0.208	0.050	3.42E-05
	8	TG.52.4	Triacylglycerol	G	T	1216	−0.084	0.021	6.08E-05
	8	TG.53.2	Triacylglycerol	G	T	1,216	−0.130	0.032	6.23E-05
	8	DG.38.5	Diacylglycerol	G	T	1,216	−0.159	0.040	6.91E-05
	8	TG.56.7	Triacylglycerol	G	T	1,216	−0.145	0.038	1.16E-04
	8	TG.52.5	Triacylglycerol	G	T	1,216	−0.125	0.033	1.39E-04
	8	TG.51.4	Triacylglycerol	G	T	1,216	−0.145	0.039	1.99E-04
	8	TG.51.5	Triacylglycerol	G	T	1,216	−0.141	0.038	2.62E-04
rs114108468	3	PE.36.4	Phosphatidylethanolamine	G	T	225	1.268	0.263	2.65E-06
	3	PE.38.4	Phosphatidylethanolamine	G	T	225	0.991	0.209	3.80E-06
	3	DG.38.5	Diacylglycerol	G	T	225	1.074	0.233	7.01E-06
	3	DG.34.2	Diacylglycerol	G	T	225	1.300	0.283	7.54E-06
	3	PE.36.1	Phosphatidylethanolamine	G	T	225	0.937	0.214	1.84E-05
	3	DG.34.1	Diacylglycerol	G	T	225	1.307	0.307	3.12E-05
	3	PE.34.2	Phosphatidylethanolamine	G	T	225	0.931	0.225	5.08E-05
	3	TG.56.1	Triacylglycerol	G	T	225	1.462	0.358	6.32E-05
	3	DG.36.3	Diacylglycerol	G	T	225	1.119	0.277	7.63E-05
	3	TG.53.2	Triacylglycerol	G	T	225	0.793	0.197	7.74E-05
	3	TG.56.5	Triacylglycerol	G	T	225	0.588	0.146	7.96E-05
	3	DG.36.2	Diacylglycerol	G	T	225	1.113	0.279	9.13E-05
	3	TG.56.4	Triacylglycerol	G	T	225	0.765	0.192	9.49E-05
	3	CE.22.6	Cholesteryl ester	G	T	225	−0.853	0.216	1.09E-04
	3	DG.34.3	Diacylglycerol	G	T	225	1.086	0.276	1.11E-04
	3	TG.56.3	Triacylglycerol	G	T	225	0.767	0.196	1.26E-04
	3	TG.56.2	Triacylglycerol	G	T	225	1.152	0.298	1.49E-04
	3	TG.53.1	Triacylglycerol	G	T	225	1.206	0.314	1.65E-04
	3	PE.38.7	Phosphatidylethanolamine	G	T	225	1.049	0.274	1.69E-04
	3	DG.36.4	Diacylglycerol	G	T	225	1.176	0.309	1.85E-04
	3	TG.58.3	Triacylglycerol	G	T	225	1.206	0.322	2.28E-04
	3	TG.51.4	Triacylglycerol	G	T	225	0.872	0.233	2.35E-04
rs16864075	3	TG.58.6	Triacylglycerol	G	A	228	0.332	0.072	6.16E-06
	3	TG.56.1	Triacylglycerol	G	A	228	0.676	0.147	7.45E-06
	3	TG.56.2	Triacylglycerol	G	A	228	0.549	0.123	1.26E-05
	3	TG.58.3	Triacylglycerol	G	A	228	0.584	0.131	1.40E-05
	3	CE.20.4	Cholesteryl ester	G	A	228	−0.175	0.042	4.69E-05
	3	TG.56.3	Triacylglycerol	G	A	228	0.326	0.080	6.97E-05
	3	CE.22.6	Cholesteryl ester	G	A	228	−0.360	0.089	7.15E-05
	3	DG.34.3	Diacylglycerol	G	A	228	0.441	0.111	9.78E-05
	3	TG.53.1	Triacylglycerol	G	A	228	0.492	0.132	2.39E-04

β, the effect estimate; CA, the allele used to estimate the effect; Chr, chromosome number; Non_CA, the other allele not used for estimation of the effect.

### Association of ΔTHR with *LPL* locus and 3q28 locus was replicated in FOS

The heritability of ΔTHR in FOS was 36%. To replicate the association of *LPL* locus with ΔTHR, using 859 nondiabetic FOS participants and a linear mixed model, we obtained ΔTHR and performed the analyses. A total of 236 SNPs (50 independent signals with *r*^2^ ≤ 0.2) within the region of 15 significant variants (19,985,951 bp−20,019,950 bp) and 314 SNPs (85 independent signals with *r*^2^ ≤ 0.2) within the region of *LPL* gene (19,901,717 bp—19,967,259 bp) were present in FOS WGS data. None of the SNPs in *LPL* locus passed the Bonferroni-corrected threshold (0.05/50 = 1e-3 and 0.05/85 = 5.88e-4). Though not reaching nominal significance (*P* = 0.279), the SNP rs79407615 G allele had the consistent negative effect (β = −0.02) on ΔTHR in FOS. In addition, a nearby rare SNP (G = 0.79%) rs117956669, 2,454 bp downstream of our sentinel variant, shows nominal significance in association with lower ΔTHR (β = −0.184; *P* = 5.9e-3). The variant rs117956669 is completely independent of rs79407615, with a negligible *r*^2^ value between them (*r*^2^ = 0.00087). We also found that a common intronic variant of *LPL*, rs112122343 (T = 5.81%; β = 0.079; *P* = 2.94e-3), is nominally significant for ΔTHR.

Similarly, we tested the association of sequenced variants that are ± 20 Kb of rs114108468 and rs16864075 with ΔTHR. Both these SNPs at 3q28 locus did not reach nominal significance in FOS. However, rs189908673 (MAF = 0.2%; β = 0.277; *P* = 1.27e-2; *r*^2^ = 0 with rs114108468) that is located 11,998 bp downstream of rs114108468 and rs113022387 (MAF = 0.45%; β = 0.1747; *P* = 4.75e-2; *r*^2^ = 0.0001 with rs16864075) 835 bp upstream of rs16864075 had a weak association with ΔTHR.

## Discussion

In our study, we characterized the genetic architecture underlying ΔTHR via two orthogonal approaches, GWAS and GWLS. In GWAS, we used a linear mixed model to account for the relatedness of 1384 LLFS participants and identified a genome-wide significant common variant of rs79407615 at chromosome 8 whose G allele is enriched in LLFS and associated with lower ΔTHR and THR. Within LLFS, this SNP is an expression quantitative trait loci for *LPL*, displaying a significant increased blood *LPL* and 14 blood lipids level with the G allele. This enriched protective G allele might be the mechanism underpinning the lower incidence of T2D in LLFS. In line with our findings, *LPL* locus (rs7015766, rs254, rs268, rs276, rs73667496, and rs55682243) has been reported regulating cross-sectional THR (4). Even though *LPL* locus (rs254, rs268, and rs276) has been reported for association with cross-sectional THR (4), the association of *LPL* locus with longitudinal ΔTHR in a healthy family cohort is novel and has not been identified before. The biological function of *LPL* is in line with our findings. *LPL* encodes an enzyme that degrades blood TG, and its deficiency causes type 1 familial dyslipidemia (21). Given elevated THR is associated with a higher risk of developing diabetes, we reasoned from our findings that elevated blood LPL within reference limits could lead to slower increase of THR (i.e., lower ΔTHR) and offers a beneficial effect in disease prevention. Conversely, nondiabetic patients with relatively higher ΔTHR and lower LPL level should be proactively managed. Quantification of ΔTHR, LPL level and measurement of blood LPL activity will be informative to screen prediabetes and provide personalized care to high-risk individuals. Preventive approaches targeting activation of LPL might be appropriate for these patients before the onset of diabetes.

In GWLS, we conducted oligogenic linkage analysis to model the association of shared genetic components in LLFS families with the similarity of ΔTHR. We found a significant novel linkage peak at 3q28 with LOD score exceeding 3. With 25 linkage-enriched families (pedigree-specific LODs >0.1), we leveraged sequence data and detected two novel variants (*EIF4A2*/*ADIPOQ*-rs114108468, *TPRG1*-rs168640750), each explaining approximately 28%∼29% of the linkage. Notably, variant rs114108468 is an expression quantitative trait loci of *EIF4A2* and *ADIPOQ* in blood, indicating their involvement in regulating ΔTHR.

Our findings are supported by multiple lines of evidence. *EIF4A2* is involved in negative regulation of RNA-directed 5′-3′ RNA polymerase activity and glucose homeostasis control by controlling specific mRNA translation and protein synthesis rate in pancreatic β cells (22). The downregulation of this gene by high glucose in rat β-INS832/13 cell supports its role as a potential candidate gene for T2D (22). *ADIPOQ* gene encodes adiponectin, which is an adipokine involved in regulation of glucose and fatty acid levels. Lower plasma level of adiponectin has reported association with IR (23) and dyslipidemia (24). Chronic administration of recombinant adiponectin to rodents improved insulin sensitivity (25). The lower levels of *EIF4A2 and ADIPOQ* in blood observed in G allele carriers of rs114108468 might be one of the molecular mechanisms underlying the accelerated increase of THR among these individuals. Based on our findings, as well as evidence by others, we proposed that strategy to increase expression of *EIF4A2* and *ADIPOQ* might be preventive for nondiabetic patients with higher ΔTHR, lower *EIF4A2*, and lower *ADIPOQ*.

The association of this locus with other IR-related traits is in line with their regulation in ΔTHR. For instance, multiple lipids were significantly associated with 3q28 locus. Several previous studies found suggestive evidence of linkage at 3q28 locus for adiponectin (26, 27, 28, 29), dementia (30), Alzheimer’s disease (31), and systolic blood pressure (32), highlighting the pleiotropic roles of this locus for multiple traits.

GWAS identified *LPL* locus, whereas GWLS with fine-mapping detected *EIF4A2*/*ADIPOQ* and *TPRG1*. The difference between these two methods arises from the distinct models they employ. GWAS estimates the effects of variants on the traits by adjusting the family relatedness using a linear mixed model. GWLS evaluates the contribution of the genetic region to traits by calculating the likelihood of nonzero additive genetic variance given the phenotypic covariance within families through a variance-component model (16). As a result, GWLS has greater power to detect rare or common causal variants inherited within families, while GWAS is more effective at identifying associations between common variants and ΔTHR that lacks the strong familial effects.

To replicate the association of rs114108468 and rs16864075 with ΔTHR, we used the 859 FOS samples from offspring examinations 5 and 7. Even though these two SNPs did not reach nominal significance in FOS, we noted two nearby rare SNPs (rs189908673: 11,998 bp downstream of rs114108468; rs113022387: 835 bp upstream of rs16864075) that are nominally significant in association with ΔTHR. This indicates that there are distinct variants within the identified locus regulating ΔTHR for FOS participants.

Despite these findings, it is important to acknowledge several limitations in our study. First, only modest effects of *LPL* locus and 3q28 locus were detected in FOS. This might be due to the difference in lifestyle and genetics across LLFS and FOS. Dietary habits and exercise intensity are well-known lifestyle factors influencing IR. However, we are unable to acquire these data, and our analyses did not account for these factors. In addition, LLFS samples were selected from exceptionally long-lived families, which might lead to selection bias with genetic heterogeneous samples differed from FOS. This is corroborated by higher levels of HDL-C and lower levels of TG across all age categories (<60, 60–80, 80–100, and >100) in LLFS than in FOS (5). Nevertheless, our results highlighted novel regulatory mechanisms for ΔTHR and nominated three potential diagnostic markers and therapeutic targets for prediabetes. Further validation of the roles of *LPL* locus as well as 3q28 locus in IR using additional studies is warranted. Moreover, the application of these candidates in clinical settings requires thorough evaluation and testing. Second, BMI might influence ΔTHR, blood transcript levels, as well as blood lipid levels. Our study did not perform an extra sensitivity analysis to investigate the effects of BMI on our results. Third, owing to the relatively low allele frequency and the constraints of small sample size, our current analysis did not reveal molecules directly linked to rs16864075. Addressing these limitations would require future investigations, leveraging an expanded LLFS offspring generation and integrating multilayer omic data, which will be instrumental in gaining a more comprehensive understanding of these genetic associations. Fourth, all LLFS samples are of European descent, limiting the generalizability of our findings to other ancestries. Validating our results using samples from ancestries beyond European descent will be crucial to address this limitation. Fifth, we cannot definitely conclude that the identified genetic variants are causal for ΔTHR without further functional experimental validation. Nonetheless, our analyses possessed several strengths. We utilized a family design, employed two orthogonal approaches, and integrated multiomics data (genomic, transcriptomic, and lipidomic).

In summary, by focusing on the genetic region under the linkage peak and utilizing the families selected for linkage, we uncovered novel variants and nominated potential genes (*EIF4A2*, *ADIPOQ*, and*TPRG1*) influencing ΔTHR. The findings add new information in better understanding of pathophysiology of IR-associated chronic diseases and processes including T2D, Alzheimer’s disease, and aging.

## Data availability

No supplemental data/tables/figures will be uploaded. All data used in this report are contained within this article.

## Conflict of interest

The authors declare that they have no conflicts of interest with the contents of this article.
